# Experiments and Observations upon the Pathological Changes Produced upon Arteries by the Ligature and by Torsion

**Published:** 1846-10-01

**Authors:** 


I. Delle Alterazioni Patologiche delle Arterie per la Legatura et la Torsione. Esperienze et Osservazioni di *Luigi Porta*, Professore di Clinica Chirurgica nell' I. R. Università di Pavia. Un vol. in 4to. grande, di pag 439, con 13 tavole in rame. Milano, tipografia di Giuseppe Bernardoni di Giovanni, 1845.

Experiments and Observations upon the Pathological Changes produced upon Arteries by the Ligature and by Torsion. By *Louis Porta*, Professor of Clinical Surgery in the University of Pavia. Large 4to, pp. 439, 13 copper-plates. Milan, 1845.

II. On Wounds of the Arteries of the Human Body, with the Treatment and Operations required for their Cure. By *G. J. Guthrie*, F.R.S. 8vo. pp. 97. London, 1846.

In the remembrance of what has been effected for the investigation and the illustration of the pathology of the arteries and for the determination of the surgical proceedings their diseases and injuries require, by means of the labours of Hunter, Desault, Scarpa, Hodgson, Jones, Bell, Cooper, and last, though not least, Guthrie and the other army surgeons, we might have been disposed to declare that no part of the domain of surgery ought to be so assured as this, and none so securely regulated by fixed and well-established principles and laws. And yet here we have, just fresh from the press, an elaborate experimental treatise upon the effects of ligature and torsion from the pen of Professor Porta, and the report of a short course of lectures upon wounds of the arteries just delivered by Mr. Guthrie, in which he censures with a just severity the retention of modes of procedure in reference to these, which the information the profession has for years been in possession of, ought long since have induced it to discard. We certainly were not aware, prior to the perusal of these Lectures, that sound surgical and anatomical principles were still so much unknown, or so little regarded, even by men of great note, as the instances cited prove to be the case. It says little for the real progress of our art that doctrines which were laid down in 1815, as the results of extensive and diversified experience, and the correctness of which has never been successfully impugned, should require reiteration in 1846, and should receive additional means of illustration from some of the instances of the consequences of neglecting them, which have occurred in the interval.

The treatise of Professor Porta is a very able performance, proving its author to be a worthy successor of the celebrated man who formerly adorned the University of Pavia. But, while testifying to the acute spirit of investigation, the felicity of exposition, and the pains-taking desire to arrive at the truth, which it manifests, we cannot conceal from ourselves the conviction that a considerable portion of it is a work of supererogation. Surely, after the elaborate investigations of Jones, Hodgson, and others, upon the effects of ligatures on the blood-vessels of animals and men, hundreds of additional experiments upon the former were scarcely called for; and indeed this portion must be looked upon rather as an elaborate demonstration or exposition of the whole case of the ligature, (or as a repetition of such demonstration in a more methodical form,) than as the receptacle of any discovery, or the herald of any improvement. The author arrives at the conclusion that the present universally adopted mode of applying the ligature is the best—a conclusion he could have as well reached had he spent a somewhat larger portion of the nine years over which these investigations have been carried in his library, instead of in the performance of numerous cruel and unsatisfactory experiments. Yes, cruel; for, surely every unnecessary experiment is a cruelty, and many, very many, of these upon the different kinds of ligatures, which universal consent has abandoned the use of, and for the re-ascertainment of points already established, must be so considered. The conclusions, too, derived from the experiments upon the blood-vessels of animals are more unsatisfactory than those which bear reference to any other portions of the economy; these tubes being gifted with very different properties in different brutes, so as to render the reasoning from analogy respecting them an unsafe procedure.

"Although," says Mr. Guthrie, "there is so general a resemblance between the arteries of man and animals, as to render them apparently similar, their structure is not exactly alike. A second cellular coat, for instance, is found between the external and middle tunics in the ox. It has not been practicable to cause an aneurism in dogs, and the apparent similarity of these vessels, with reference to the effects which may result from injury or disease, cannot be depended upon: nor can any confidence be granted to the numerous experiments which have been very cruelly made upon animals with the view of elucidating the various processes which occur in man."

But while protesting against this part of the work as uncalled-for, though ably executed, we admit that other portions relating to the demonstration of the collateral circulation are more original in their anatomical demonstrations, though still leading to no novel conclusion or improvement in practice. The numerous facts cited from his own practice and that of various celebrated surgeons are however valuable; and altogether the work is highly useful to those (as we suppose is the case with many in Italy) who have not access to the other sources of information we have referred to. A brief analysis of its contents may also be interesting to our readers.

In the First Chapter the *Anatomy* of the *Arteries* is treated of. The author, like other anatomists, describing these as consisting of three coats, the cellular, fibrous, and inner. Blood-vessels, which are found abundantly supplying the outer tunic, become smaller and smaller, and are gradually lost as they penetrate the middle one, so that they cannot in any wise be traced upon the innermost.* The nerves and lymphatics do not penetrate beyond the cellular coat. The *external coat* is then an eminently vascular membrane, and pathological changes in man, as well as experiments in brutes, alike prove it to be the seat of the principal vital phenomena and organic metamorphoses which take place in the arteries. The *middle* one resembles tendinous or aponeurotic structure, and seems destined chiefly to give form and resistance to the vessel, and to preserve its calibre without presenting an obstacle to the impelling action of the heart. The

* There is a discrepancy in this statement of the author compared with that of the concluding sentence of the paragraph.

*innermost* tunic is composed of a fragile, coriaceous pellicle—an arterial epithelium. This epithelium being lined with a serous membrane, is pretty vascular, but it is by no means so prone to take on inflammatory action as represented by some pathologists, although inflammation of the proper tunics of these vessels, which is denied by others, and is really of rare occurrence, may yet undoubtedly take place.

In the Second Chapter the following questions are discussed :—*What becomes of a ligature attached to an artery? Does the material of which it is composed influence the results of the operation? What is the most eligible material?* Four descriptions of ligature were tried in the 300 experiments performed to illustrate these points. Catgut was employed 80 times, silk 120, thread 60, and horse-hair 40 times. The results are detailed at great length, but we need only advert to the general conclusions, viz. that of the 300 ligatures, 64 disappeared in periods varying from one day to two years, and 236 remained : of these last, 29 were found amid a lymphatico-cellular tumefaction, 60 within the intermediate cord connecting the two ends of the artery, 67 enveloped in common cellular tissue, 54 enclosed in a true cyst, and 26 in the midst of suppuration. Our author considers the 64 which disappeared were assimilated (or more correctly speaking absorbed), and found a much larger proportion of the *catgut* ligatures underwent this process than those of any other material. Those results observed in animals differ from those observed in man by the much greater frequency with which the ligature excites the adhesive process in the former by which it is retained, and the suppurative process in the latter by which it is discharged. Signor Porta enquires whether these ligatures, which after amputation have had their ends cut short, as recommended by Lawrence (but seldom put into force by him now we imagine), and as treated by himself both after amputation and the operation of aneurism, and which are sometimes retained after the wound has united and never discharged by the suppuration, do not become dissolved or encysted. It may be so, but seeing how very rarely such a result can be calculated upon, we think so generally an unsuccessful practice should be discountenanced.

In the Third Chapter the author discusses the various modes of applying the ligature and performing torsion, and the pathological changes which are produced upon the arteries by these.

1. *On the Application of the Ligature.*—Four kinds of ligature are described, viz. the common or circular, the temporary, the mediate, and the double.

(A.) *The Circular Ligature.*—With this ligature formed of various materials, the ends being cut off and union sought to be obtained, 140 experiments were tried on various arteries of different animals. From these the ordinarily described mechanical effects, division of the proper tunics, strangulation and approximation of the cellular tunic, and the formation of a coagulum on each side the thread, resulted. The obliteration of the vessel is due to the subsequent inflammation, which is so much more commonly of an adhesive character in animals than in man, that in 140 experiments the ligature was only discharged with suppuration 19 times. In man a mixed process, termed by the author plastico-suppurative, takes place.

" Operating by whatever mode we will, we can never hope to obtain in surgery the happy results derived from tying arteries in animals; but, judging from analogy, and from known data, I believe that when the ligature is applied in man with greater delicacy than it has hitherto been, choosing the most homogeneous material and the most simple procedure, respecting the cellular matrix of the vessel, cutting off the ends of the ligature, closing up the wound, avoiding the proximity of the aneurism, and enjoining the greatest quietude to the patient, we shall obtain adhesion much oftener; and that when, in spite of our efforts, suppuration does occur, it will be circumscribed, and will not impede the plastic exudation which closes up the mouths of vessels, and prevents those fatal accidents which, so exceedingly rare in animals, have so frequently endangered the life of man."

As Mr. Guthrie's account of the effect of the ligature upon the vessels differs from that usually given, we will here extract it.

" The inner and middle coats are not only divided, but the inner one particularly appears curled inwards on itself, so that the cut-edge of one half or side is not applied to its fellow in the usual way of two surfaces, but by curling inwards meets its opponent on every point of a circle, and in this way forms a barrier inside that of the external coat, which is tied around it by the ligature: so that, in fact, when a small ligature is firmly tied, its direct pressure is not applied to the inner coats, which have been divided, and have curled away from it, but to the outer one, which is in consequence of that pressure made to ulcerate or slough, which processes could scarcely fail to take place also in the other coats, if they were subjected to pressure in a similar manner. The cut-edges, being from this provision of nature perfectly free, are capable of taking on the process of inflammation, which stops at the adhesive stage. This they do by the effusion of lymph or fibrin, both within and without, to a greater or less extent as the case may require. The outer coat of the artery must either yield by ulceration or sloughing, or the ligature must remain until it is decomposed and destroyed. The artery usually yields by sloughing, and the ligature is left at liberty by the ulceration which takes place in the sound part of the artery immediately above and below the part strangulated by the ligature, and which part is frequently brought away in the noose. The artery does not always yield by sloughing, particularly if it is a large one, and the ligature has been thick and soft. In this case, a part of the outer coat, from its folding or plaiting under the ligature, seems to escape that degree of pressure necessary to destroy it, and when the remaining part yields, it remains entire, and is only removed by a subsequent process of ulceration, occasioned by its irritation, as an extraneous body. I have had the opportunity and misfortune of examining great numbers of stumps after amputation and death, and I have seen this occur in so many instances as to leave no doubt of the fact. In these cases, the external coat could not close around the inner ones; and this shows that they are capable of forming an effectual barrier without it, although it materially assists in giving greater strength to the cicatrix by the effusion of fibrin, which takes place within, without, and around.

" Whilst this process is going on without, and at the very extremity of the artery, the vessel is gradually contracted above it, and its coats become more or less inflamed, soft, and vascular. The inner coat is seen to be wrinkled transversely, and a small coagulum of blood is formed within it. This sometimes completely fills the artery, but it is more common for a small tapering coagulum to be formed, adhering by its base to the extremity of the inner coat. My observations have led me to believe that a coagulum is not absolutely necessary to the permanent closure of an artery, although it certainly assists in maintaining it."

Although an artery is said to contract until the next collateral branch, Mr. Guthrie has met with several cases in which the principal vessel has continued pervious below such. Again, it is an error to suppose that a collateral branch immediately above the ligature always interferes with the closure of the canal. It sometimes may prevent it, and generally delays consolidation. "I have so often seen large arteries heal after division close to the giving off of a considerable branch, that I consider them always capable of doing so if they are naturally sound. The power which suppresses hæmorrhage in a bleeding artery, resides at the very extremity of the vessel itself." *Ligatures* should be round, and as small as consistent with strength. The surgeon should acquire a knowledge of the strength with which they require to be tied on the dead body, and he will generally find that he has estimated the necessary force too highly "A ligature composed of one strong thread of dentist's silk, well waxed, is sufficiently firm for the largest artery."

(B.) *The Temporary Ligature.*—With this 50 experiments have been performed, leaving it on the vessel for periods varying from a few minutes to several hours. The proper tunics of the vessel are divided, and slight inflammatory action induced. By the third or fourth week the artery has entirely resumed its normal condition, scarcely any thickening of the cellular membrane even remaining. The blood never ceased flowing through the vessel, except in 6 or 7 cases, in which coagula, and in 5 or 6 others, in which lymph, partially or entirely, but temporarily, obstructed its course. These results being so different from those produced by the experiments of Travers and other English writers, 24 other additional experiments were resorted to, in which two, three, or four ligatures were left on the vessels very near to each other for spaces of time varying from 2 to 12 hours. In some of these, coagula obstructing the vessel formed, but in the majority no such obliteration took place.

(C.) The *Mediate Ligature* so warmly advocated by Scarpa, and still employed by M. Roux, was the subject of 120 experiments, in 35 the ligature being permanent and in 85 temporary. The *permanent mediate ligature* (a small waxen cylinder being interposed between the thread and the artery) induced four changes in the vessel: 1, an arteritis of the cellular membrane; 2, an erosion of the proper tunic, which commenced from the 2nd to the 7th day, at the point over which the little cylinder was applied; 3, the section of the artery took place sometimes in 60 or 70 hours, but generally not before the commencement of the 2nd week. The section was followed by a retraction of the ends of the artery which were divided by an interval of from 2 to 6 lines, and by the separation of the cellular deposit into two portions, so that the extremities of the vessel were only now constituted by the internal coagulum. 4. The separate cicatrization of the two ends of the vessel. In the 85 instances of *temporary mediate ligature*, (the thread being retained on from 1 to 5 days), in 33 the vessels had undergone no change whatever, and in 21 they were scarcely eroded. In 9 they were eroded and cicatrized, in 16 divided right through, and in 6 reduced to a cellular cord. The vessel was completely obliterated in 61 cases, 34 times by reason of the presence of a coagulum, 11 by deposition of lymph, and 16 times by the section of the arterial walls. In the other 24 cases the arteries remained permeable. In 7 there was hæmorrhage.

(D.) In 45 cases the *Double* ligature (the threads being applied at a few lines distance from each other) was tried, in 35 of which, simple section of the intervening artery was performed, and in 10 a portion of the vessel of varying length was removed. The mechanical alterations produced in the vessel were the same as in the case of the circular ligature, *plus* the excision of the artery, the ecchymosis and the retraction and coarctation of the ends of the vessel. The subsequent inflammation of the cellular tissue gives rise to a considerable deposit of plastic lymph which is modelled into a cylinder, completing the continuity of the vessel, and being sometimes impervious, and at others containing a cavity filled with sanguinolent serum. It is at first of a larger diameter than the artery it replaces, but gradually atrophies into a thin fibrous cord, and eventually becomes absorbed. If only one or two centimetres be removed from the artery, it comports itself as just described; but, if more be removed, its two ends cicatrize separately. In three cases hæmorrhage was observed, which was never the case in the experiments with the circular ligature.

The author concludes this section by an estimate of these various measures.

"The *temporary* ligature must be looked upon in the light of a simple experiment, destined always to remain fruitless by reason of its inefficiency. The *permanent mediate* ligature, once recommended by prejudice, should be consigned to the just neglect which has so long attended it. As to the *temporary mediate* ligature, so seducing at first sight, we may declare it, from the preceding demonstration, to be a difficult, complicated, and uncertain method. To these faults the *double* ligature adds violence, complexity of procedure, occasional impossibility of execution, copious suppuration, and a greater liability to hæmorrhage. There only remains then the *circular* ligature to which good sense and general custom had accorded a preference that science approves and justifies. By a fortunate combination, all the data to which we are accustomed in surgery to have recourse for the appreciation of a method are propitious to this. The simplicity of the mechanism of the operation, the little irritation it gives rise to, the regularity and mildness of the process by means of which the artery becomes obliterated, and especially the promptitude of the cure and the large proportion of success, are the prerogatives which render, and will always render, this superior to all other methods. The circular ligature is not faultless, and reverses may occur under its use, but for its choice it suffices to know that it is the most easy and most useful procedure. The idea of closing an open artery with a circular thread seems so natural, that it must have presented itself to the first operator and signalised the origin of the operation. The disasters which in the course of time attended its employment, and ignorance of their true causes, suggested a multitude of procedures, which, received without criticism, have come down with various vicissitudes, even to our own times. But, at the present period, science and practical observation have so thoroughly examined all these methods, that the danger of following an error upon the authority of others, or fostered by a blind empiricism, is removed."

Section 2. *On the Torsion of Arteries.*—The author is a warm admirer of this practice, which has met with so little encouragement in England. Torsion produces both mechanical and organic effects. To the first we refer the circular laceration and the introflexion of the proper tunics of the vessel which sometimes takes place, the strangulation of the cellular tunic, and the formation of the internal clot. The second are analogous to those which result from ligature. We are here presented with the results of 60 experiments made on animals, and, what is of far more importance, the relation of the author's experience of this means applied to man. He has submitted to torsion during the course of the last nine years, both in public and private practice, a very great number of arteries, such as the occipital, temporal, maxillary, superior thyroid, external pudendal, spermatic, digital, and some branches of the subclavian, and indeed all the arteries of the fourth and fifth rank when wounded at the surface of the body, and always with entire success. In regard to those of the extremities, he possesses accounts of 65 cases of torsion after amputation or disarticulations performed by him, and of those of the upper extremity all succeeded except two, in which it was necessary to recur to the ligature; and of 23 cases of torsion of the femoral and popliteal, the ligature was subsequently required in four instances. "It is not unfrequent, however," he observes, after describing the rapidity with which the proceeding may be conducted, "for a first torsion to fail on some arteries, either because they have not been sufficiently twisted, or the vessel having been ineffectually separated from its connections, the laceration of its proper tunics has not taken place. We must repeat the torsion a second or a third time, until we do succeed." After some observations upon the ulceration of the twisted portion, and the partial rupture of the cellular tunic at the borders of the seat of torsion, accidents which, however rare, may yet occur, and give rise to a hæmorrhage only to be restrained by the ligature, the author thus concludes:

"Torsion should be considered simply as succedaneous to the ligature in wounds of the arteries, and with this indication we may, without hesitation, pronounce it a preferable and an entirely safe operation for vessels of a moderate size, and in the majority of cases, if not in all, a successful one for the larger vessels of the extremities."

Chap. 4. *On the Collateral Circulation established after the Obliteration of the Arterial Trunks.*—This is a most elaborate and able chapter, in which Professor Porta, by the aid of experiments on animals and observation on man, exhibits the most complete view of the collateral circulation with which we are acquainted. He treats successively of the direct and the indirect collateral circulation.

(A.) *The Direct Collateral Circulation.*—Our want of space quite prohibits our attempting to follow him in his demonstration of this; and it must suffice to observe that he applies the term to the enlargement of prior existing, and the production of new capillary vessels, constituting an immense net-work of *vasa vasorum* surrounding the two ends of the vessel and the newly-deposited lymph, and springing from the internal coagula as a matrix. At first these distended vessels exist in countless numbers, but, as the phlogosis becomes subdued, some of the more direct and larger branches only acquire and retain a great size, the others resuming their former condition. The author's observations upon direct anastomosis have been carried on by means of experiments on dogs killed at periods varying from a week to $2\frac{1}{2}$ years after the ligatures; but in certain necroscopies he has had the opportunity of making upon individuals, who have died some years after the tying of large arteries, the same system of direct collateral circulation was visible enough.

(B.) *The Indirect Collateral Circulation* is accomplished through two different routes, the *deep-seated* or *muscular*, and the *superficial* or *sub-cutaneous anastomoses*. The author details with great minuteness the changes which are operated in the anastomotic system from the first day to the third year after the application of the ligature upon all the principal vessels, as the femoral, carotid, iliac, &c. It is obviously impossible that we should follow him through this elaborate demonstration; but we may advert to his observations upon the alterations which the general arterial system undergoes subsequently to the application of the ligature upon a principal central trunk. These are exhibited in three distinct places, viz., the trunk that has been tied, the collateral trunk, and the anastomosing branches. 1. *The ligatured part*. The two ends of the divided artery gradually diminish in size, although in corresponding points they preserve a certain calibre for the reception of the anastomoses destined to maintain the circulation. The *vasa efferentia* of the inferior portion, converted now into *vasa inferentia*, preserve its natural calibre from their origin below, and restore to it somewhat later a part at least of its natural pulsation; which proves that the indirect flow of blood by the anastomosing branches is sufficiently abundant from the earliest period to maintain the movement and capacity of the inferior portion. 2. The metamorphosis to which the *collateral trunks* are subjected, consists in their amplification. The superior branches are gradually enlarged to the extent of being able to supply the place of the obliterated trunk, conveying as much blood, or nearly so, as had passed by it. Nevertheless, we cannot demonstrate upon the dead body for some weeks an unusual enlargement, and permanent dilatation is of so gradual a formation that it does not become completely developed until after the expiration of several months. But, by reason of the dilatability of the arteries under the force of the heart's impulse, or by means of an organico-vital action exerted upon the anastomotic ramifications of the smaller branches, a distension, which cannot be exhibited after death, yet does occur during life. The dilatation is much more irregular in the collateral branches connected with the lower portion of the artery than in those arising from the upper. When the collateral circulation is matured, the sum of the efferential and inferential branches is about equal to the calibre of the lost artery, and the change operated in them, upon the occasion of the use of the ligature, is permanent, although, after the death of the individual, we may find some of them not larger than in the limb which had not been operated upon, either because their dilatation had not yet become organic and permanent, or that they had taken none or a very slight part in the new collateral circulation. 3. The *anastomozing branches* undergo changes as respects their calibre, form, and structure. Their unusual dilatation is observed from the earliest period by the aid of injections. Throughout the anastomozing system the medium branches are first seen in a few days to become turgid, then the secondary ramifications, and, lastly, the lateral trunks, which communicate with the two extremities of the obliterated artery. Within the first week the anastomotic branches double their capacity and change their form. It is the anastomotic arches which occupy the centres of the muscles and proceed directly towards the distal end of the artery that first enlarge, and then those which pursue a longer and more tortuous course towards the periphery. The entire anastomotic system does not acquire its full development for several months or even a year. Observed at a more remote epoch, no ulterior progress is noted, and vessels of three sizes are then discernible. The larger have a calibre of two millimetres; the medium of one, and the smallest little more than that of the ordinary capillaries. The deep anastomotic arches, while enlarging, take on very different forms, being sometimes simple, at others complex, rectilinear, circular, elipsoid, rhomboid, &c.; sometimes they are twisted into small knots, resembling tumours, purses, &c., and then regain their regular form. At first, the walls of these vessels are soft, transparent, and fragile, but eventually they become dense and resisting. The vessels, in enlarging, may become twisted upon themselves in a serpentine or convoluted manner, and very small lateral branches, which perhaps may have pre-existed in a rudimentary state, are seen to spring forth, and intercommunicating, describe simple or complex causes, and return at various distances to the principal arch, thus increasing the vascular interlacement and the facility of communication.

Professor Porta believes that these facts observed so constantly, positively, and clearly as regards the system of indirect anastomoses in animals, must serve as a foundation for any true theory upon the subject, and that, if the same experiments could be performed on man, very similar results would probably be observed. He relates the history of the pathological changes in the arterial system after the application of ligatures on some of the great arteries observed by himself and others, and, comparing these with the inductions drawn from experiments upon animals, he arrives at the conclusion that the analogy is very striking, and that the sole differences are the following:

"1. In animals they are generally the same vessels which form the collateral circulation, and these always undergo the same alterations. In man, such constancy is not observed; sometimes the branches of one side, and sometimes those of the opposite, form the principal communications. 2. In the former, when the principal trunk is closed at one point, the collateral branches conduct the stream of blood to the same point of the inferior portion of the artery; but in man, on account of the presence of an aneurism which has required an operation, the trunk becomes obliterated over a long track, or in several distinct places; whence, instead of one there is often formed a chain of two or three anastomotic rings along the whole course of the limb. This is seen especially at the hip, where the blood of the circumflex and perforating arteries only reaches the leg through the articular and recurrent tibials. 3. Comparing the pathological observations with our zootomic experiments, it results that the blood, which at first flowed through numerous small vessels, is afterwards included in a few large channels, to which the collateral circulation is circumscribed. 4. Just as the phenomena of the collateral circulation are simple and constant in animals they appear in man complicated, anomalous and versatile. It is possible and occurs in the human body, according to the pathological condition of the limb or the individual, that the arterial system, impeded in its natural direction, may change in different cases the route of the blood, and may limit the collateral circulation to a few principal vessels. But the fact, in animals, has been rendered clear by repeated experiments and dissections; while, in man, it is vague and undetermined, in consequence of the uncertainty of the observations which support it."

Mr. Guthrie also offers some interesting observations upon the *collateral circulation.*

"Two distinct kinds of collateral circulation are at present acknowledged: one by direct large communicating arteries; the other, through the indirect medium of the capillary vessels, inosculating with each other. Where the direct communicating arteries exist, little subsequent change takes place in them. It is otherwise with the indirect capillary vessels. When the radial or ulnar artery is divided in the hand, the blood will not only readily flow from each end of the vessel, but equally red and arterial from both; the communication being through direct arterial branches from one vessel to the other. It will be also red and arterial if the division take place at the wrist; and may be so in the brachial. But if the femoral, in the lower part of the thigh, be wounded, the colour of the blood issuing from the lower end of the artery, if any issue at all, will be venous. It is so, because it has been obtained from the capillary arteries, which, in this case being empty, receive blood by regurgitation from the veins, the valves of which, when present, do not prevent its reflex course. If a limb be injected and carefully dissected four or five days after a ligature has been placed during life high up on the principal trunk, the capillary vessels will be seen to be well injected; but few or none will be found large enough to admit of their inosculation being traced throughout. If another limb be injected, and dissected some sixty days after the ligature has been applied, a difference will be distinctly observed between the two preparations. In the latter, the capillaries will not appear to be so fully injected, but several larger and more tortuous vessels will be found in situations where they were not expected to exist; and the anastomoses of these one with another, and generally by arches, may be traced to their communication with the principal trunk, both above and below the obliterated parts. If an incision were made in the nearest pervious portion of the lower part of an artery of a person who had undergone this operation, arterial blood would issue from it. The communication would have become directly communicating branches, and the capillaries would have returned to their accustomed duties. * * * * * * * * It is asserted by some sanguine supporters of the all-powerful influence of the collateral circulation, that it is sufficient at all times, and under all natural circumstances, to maintain the life of the extremity. The practice of the Peninsular war proved the fallacy of this opinion in too many instances, to admit of any doubt of its inadequacy to do so in the lower extremity, after the division of the femoral artery, under ordinary circumstances."

(c.) *On the Accidents which may accompany the establishment of the Collateral Circulation.*—Professor Porta considers those, which arise from an excess or deficiency of the circulating fluid consequent upon the application of a ligature. To the former he refers plethora, inflammation, and the relapse of aneurism: to the latter, paralysis, gangrene, and atrophy of the limb operated upon. The accidents arising from *plethora* or *inflammation* may exhibit themselves by their proper symptoms, either in the part affected or in some distant locality. The brain, according to the author, is a very common seat of their manifestation, and thus, of eleven cases of ligature of the carotid, five offered evident signs of cerebral plethora requiring the detraction of blood. All who are familiar with the history of this operation must be aware of the frequency with which encephalic disturbance, or even disease, has followed its performance; but it is quite new to us, and not very intelligible, that cutting off a large supply of blood to the brain should induce a state of plethora of that organ. Ligatures of other large arteries, Dr. Porta continues, give rise to similar consequences; and inflammations of the coverings or the viscera of the chest and abdomen have not unfrequently been induced; and severe inflammation of the limb operated upon is less common than that of the organs now alluded to.

"The other accident arising from the exuberance of the collateral circulation and the excessive dilatation of the anastomosing branches, is the reproduction of the pulsating aneurism. This has been observed in the neck, the hip, and in all the limbs. Oftentimes it is only temporary, because the blood having lost, during its course through the winding anastomosing vessels, the impulse that conveyed it from the heart, is no longer enabled to prevent the coagulation in the tumour. Nevertheless, it suggests to practitioners the necessity of retaining the limb and the patient himself in a state of quietude, and to ensure, sometimes with the aid of antiphlogistics, cold, or compression, the exit of the ligature. In some cases, the natural anastomoses are so large and direct, or dilate themselves with such rapidity, that they retard or prevent the coagulation, and thus impede or prevent the success of the operation."

Passing now to accidents of an opposite description, the author observes that *paralysis* and *atrophy* may simultaneously or separately follow the ligature of the artery. The nervous system presiding over the other parts of the economy receives its nutriment from the circulating system, and whatever diminishes or interrupts the course of this latter, must weaken or suspend the actions of the nervo-muscular system, sensation, and motion. In animals, paraplegia is observed to result from ligature of the aorta, and in man, paralysis of the limbs has followed the tying their principal blood-vessels. Several examples after the ligature of the humoral artery are cited. At other times, atrophy of the limb follows as a sequel to paralysis, or it may exist without any prior affection of the nervous system, owing to a paucity of nutrition after the obliteration of the principal vessel. Several cases from his own and other's practice are cited in proof.

Instances of *gangrene* supervening upon ligature of an artery are, however, far more frequent in man than those of paralysis or atrophy. "Of 600 cases of ligature of the large arteries on record, gangrene has occurred in 50. Of 132 operations in the carotids it occurred in 1; of 150 cases of ligature of the innominata, subclavian, axillary and humoral, in 7; and of 302 operations in the lower extremities, in 42." As to the superior extremity, Signor Porta believes that, unless some grave complication existed, such as the severe nature of the wound, the bad performance of the operation, hæmorrhage, affection of the chest, the inclusion of nerves in the ligature, enormous ecchymoses, gastric fever, phlegmon, &c., &c., we should perhaps never meet with such an accident. Its occurrence in the lower extremity is favoured by the greater distance from the centre of circulation, the size of the limb in proportion to the body, the small size and circumvolutions of the anastomoses, the disposition of popliteal aneurism which easily compresses and annihilates the lateral arterial radicles. "In consequence of these dispositions we almost always find, after the ligature of the iliac or femoral arteries in man, there are present, during the first days, symptoms of paralysis, formication, chilliness, and heaviness of the limb, which are sometimes followed by gangrene." The synoptical table of 600 operations, undertaken by surgeons of various nations from the time of John Hunter to the end of the year 1843, is a valuable statistical document.

In taking leave of Dr. Porta's work, we beg again to express our admiration of the patient industry and philosophical spirit of enquiry with which he has pursued and attained the object in view; although, as we before observed, we should have been better pleased had he caused less animal suffering by abstaining from many experiments,* the repetition of which was uncalled for, and has led to no novel conclusions.

Mr. Guthrie's Lectures are full of valuable facts and important critical observations, condemnatory chiefly of performing the operations for wounded arteries which are only fitted for them when in their aneurismal condition. One hundred and thirty already-published cases are briefly detailed and commented upon, furnishing the student and practitioner with some excellent precepts and cautionary remarks. Although the extract is a long one, we cannot forbear laying before our readers Mr. Guthrie's

*General Conclusions.*

"1. The Hunterian operation for the cure of an aneurism is not applicable to the treatment of a wounded artery, inasmuch as the wound of the artery communicates with the external parts, and nothing intervenes to prevent blood flowing from the wound in its side, or from its cut extremities.

"2. When a large artery is divided and bleeds, the wound should be enlarged if necessary, and a ligature placed on both the divided ends; but if the artery be only injured and not quite divided, the ligatures should be applied one immediately above, the other below the injured part. The artery may or may not be then cut across, at the pleasure of the operator, but the limb or part should be placed in a relaxed position. A bandage should not be applied, and the edges of the wound should be simply brought together by adhesive plasters, which do not extend completely round the limb.

"3. No operation is to be performed on any artery unless it bleeds at the moment of its performance, inasmuch as hemorrhage once suppressed may never return.

"4. The intervention of muscular fibres, or of whole muscles, is not a sufficient reason for tying the artery at a distant part. They must be divided, if it be possible, to the extent required for a due exposure of the injured artery and its accompanying veins and nerves.

"5. If the wound pass indirectly to the principal artery, from the back of the thigh for instance to the femoral artery in front, or from the outside of the arm to the humeral artery on the inside, the surgeon may (on satisfying himself of the part likely to be injured, by the introduction of a probe) cut down on the vessel opposite the part supposed to be wounded, by the most simple and approved method. When the artery is exposed, the probe will point out the spot

---

* We were sorry to observe, in an article in a recent number of the *Lancet* (July 18), written with the excellent intention of inciting students to a more philosophical study of their profession than now prevails in this age of manuals and vade-mecums, a recommendation that they should engage in the performance of experiments as a means of awakening their energies and exciting their attention. "One good experiment," it is said, "is worth loads of paper and pseudo-physiology." "A properly chosen experiment" is also recommended as part of the means of testing the candidate's ability.

at which the vessel has in all probability been wounded. Pressure made below this spot on the artery, will cause it to be distended and to bleed, if the flow of blood be not prevented from above: the artery is then to be secured by two ligatures, and the lower one should if possible be applied first.

" 6. The tourniquet should never be used in an operation for aneurism or for a wounded artery. Compression by the hand in the course of the wounded vessel is allowable.

" 7. The blood from the upper end of a divided artery, or that nearest the heart, is of a scarlet arterial colour.

" 10. The blood from the lower end of a divided artery, or that which is furthest from the heart, is of a dark or venous colour, when it happens to flow immediately after the division of the vessel. At a subsequent period it may assume more of the colour of arterial blood, but it rarely does so for several days after the receipt of the injury, and always flows, or at least until a very late period, in a continued stream.

" 11. This regurgitation or flow of blood from the lower end of a divided artery is a favourable sign, inasmuch as it shows that the collateral circulation will probably be sufficient to maintain the life of the extremity.

" 12. The collateral circulation is in almost every instance capable of maintaining the life of the upper extremity when the axillary artery is divided, and the colour of the blood which flows from the end of the artery, on its being divided, is not always as dark as in the lower extremity, and it sooner resumes its arterial colour.

" 13. The collateral circulation is not always capable of maintaining the life of the limb when the femoral artery is injured. The best assistance which art can give is to rub the foot and leg in the gentlest manner, between the hands of one or two strong young women, for several hours, or even for the first three or four days; relaxing this process very little, even during sleep. When the vein is divided at the same time, or rendered impervious, the limb usually mortifies.

" 14. The collateral circulation is sufficient to maintain the life of an extremity in almost every case in which an aneurism has existed for eight or ten weeks, although it may be incapable of doing this if the principal artery have been suddenly divided, without any previous disease having existed in the part.

" 15. The theory and the operation for aneurism are never to be applied to the treatment of a wounded artery, which has caused a diffused or circumscribed aneurism, *whilst the external wound communicates with the artery,* unless it be impossible or impracticable to tie the bleeding vessel.

" 16. When an artery has been wounded, and the external opening has healed for weeks or months, so as to give rise to a diffused or circumscribed aneurism, it may be treated according to the theory of aneurism occurring from an internal cause, if the case will permit it without danger; although with this difference, that as the artery is sound the operation may be performed close to the tumor. If any doubt exist as to the capability of the collateral circulation to support the life of the lower extremity, after the external iliac has been secured by ligature, the operation should be performed at the injured part by opening the swelling and enlarging the wound, as in the case of a wounded artery.

" 17. When a circumscribed or diffused aneurism which has formed after a wound has been opened, whether by accident or design, it is placed in the situation of a wounded artery, and should be treated as such. If the aneurism has arisen from disease of the vessel, and the wound or opening into it cannot be permanently closed, the limb is in a worse state than if the artery had been wounded by accident; because a ligature or ligatures placed on a diseased artery are little likely to be successful. They are liable to all the difficulties and inconveniences attendant on the old operation for aneurism. If a case of the kind should occur in a popliteal or femoral aneurism, situated at or below where the artery passes between the triceps and the bone, amputation, if it can be done low down, will be the best remedy. If the aneurism should have formed higher up, and the opening can be closed with any prospect of its healing, a ligature may be placed upon the artery above it; but on the recurrence of hemorrhage which cannot be restrained by moderate pressure, the artery must be tied below, or recourse had to amputation. It is, however, to be observed, that amputation under these circumstances, when resorted to as a third operation, rarely succeeds.

" 18. When an artery is wounded with a simple fracture of a bone, or with a comminuted fracture of smaller bones, with an external communicating opening, both ends of the artery should be secured, and the limb treated in the usual manner.

" 19. When the bone broken is the femur, and the artery divided is the femoral artery, the operation of amputation will generally be advisable. It will always be so if the fracture is a comminuted one, or the shaft of the bone is extensively splintered.

" 20. When the broken bone injures the artery and gives rise to an aneurism, the treatment is to be first of the fracture and then of the aneurism, as soon as circumstances render it advisable or necessary to have recourse to the operation for aneurism; which can only be after time has been given for the collateral branches to enlarge, so as to maintain the life of the limb.

" 19. When mortification takes place in addition to, or as a consequence of a wounded artery, amputation should be had recourse to forthwith.

" 20. The place of operation should be in almost all cases at the seat of the original wound; but there may be an exception, viz.

" 21. When, for instance, the injury has been a mere cut, just sufficient to divide the artery and vein immediately below Poupart's ligament, and mortification of the foot supervenes, amputation should then be performed below the knee, or at the part where the mortification more usually stops for a time.

" This rule is founded on the observation, that great efforts are made by Nature to arrest mortification a little below the knee. Sometimes they succeed; when they fail, death is almost inevitable. The advice to amputate at this part is founded on the fact of its being infinitely less dangerous, when done there, than on the thigh, independently of saving a joint.

" 22. When mortification has *continued for several days*, and is spreading without having once stopped, the constitution of the patient being implicated as marked by fever, amputation should not be performed until the mortification has been arrested and the line of separation has been well formed. In many cases, where there is great weakness or of irritability of constitution, it will be advisable to defer the operation to a later period, particularly if there be hope of the patient's becoming stronger and more tranquil.

" 23. If the mortification has once stopped and then begins again to spread, it will never again cease to extend, and amputation may give some chance of life.

" 24. Amputation of the arm should never be had recourse to, in consequence of a wound of the axillary artery, unless mortification takes place.

" 25. When mortification takes place after the operation for aneurism, the surgeon must be guided by the state of the patient's constitution, in resorting to or refraining from amputation.

" 26. When hemorrhage occurs from the surface of a stump, the artery should be tied at the part from which the blood comes in the first instance, if it can be easily done. If this should not suffice, the artery must be tied higher up, just at such distance as will afford a fair hope of its not having been affected by the derangement of the stump, which has led to the failure of consolidation in the extremity of the artery; yet not too high to admit of the junction of any large collateral branches. If the bleeding proceeds from several small vessels, and cannot be arrested, the principal trunk should be tied immediately above the diseased part, and the patient removed to a purer atmosphere, without which an operation rarely succeeds in any case.

"27. When an aneurismal tumor mortifies, it is unnecessary and improper to tie the artery above the tumor, because it will be obliterated if the mortification be arrested by the efforts of nature, which the operation may interfere with, and even prevent, whilst, if the mortification spreads, it will be a matter of supererogation, and only hasten the patient's dissolution. When an aneurism inflames, is opened by ulceration, and bleeds profusely, so as not to be arrested, it is a proper case for amputation, if such an operation can be performed." P. 85.

